# Mothers’ Parenting Stress and Neighborhood Characteristics in Early Childhood (Ages 0–4)

**DOI:** 10.3390/ijerph18052648

**Published:** 2021-03-05

**Authors:** Eun Jung Kim, Min Jung Cho, Mi Jeong Kim

**Affiliations:** 1 School of Architecture, Hanyang University, Seoul 04763, Korea; ejkim82@hanyang.ac.kr; 2 Faculty of Governance and Global Affairs, Leiden University College, 2595 DG The Hague, The Netherlands; m.j.cho@luc.leidenuniv.nl

**Keywords:** neighborhood characteristics, mother’s parenting stress, early childhood

## Abstract

Neighborhood characteristics are important when raising children. Traditional approaches to parental stress research have focused on the impacts of daily hassles and how individual factors, such as children’s temperament, family resources, and social support from friends and family reduce or exacerbate parental stress. There have been few studies on neighborhood characteristics and parental stress, and even fewer studies have examined the association longitudinally. The goal of the present study was to explore the association between mothers’ parental stress and neighborhood characteristics longitudinally across early childhood (ages 0–4). Using the 2008–2012 Panel Study on Korean Children, we followed 1536 mothers. The results showed that mothers’ parenting stress was highest when children were aged two to three, and neighborhood characteristics had significant associations with parenting stress. The study indicated that mothers who reported that their neighborhoods had sufficient childcare facilities, were convenient to access public recreational and cultural facilities, and those who reported that their neighborhoods were good places to raise children, exhibited significantly lower levels of parenting stress. Further, the effects of neighborhoods on mothers’ parenting stress were greatest when children were aged one and four. Hence, such findings should be incorporated when designing and developing communities.

## 1. Introduction

Neighborhood characteristics are important when raising children [1,2]. The availability of childcare centers, the provision of friendly and safe neighborhoods, and proximity to recreational and cultural facilities, such as playgrounds, parks, and public libraries, directly and indirectly influence family outcomes, such as child maltreatment [3,4], juvenile delinquency [5], and mothers’ psychological distress [6].

Yet, there have been relatively few studies on neighborhood characteristics on family outcomes, especially in relation to parenting stress. Traditional approaches to parental stress research have focused on the impacts of daily hassles, such as the stress of meeting children’s needs, monitoring them, and dealing with their resistance [7,8] and how individual factors, such as children’s temperament, family resources, and social support from friends and family, reduce or exacerbate parental stress [9]. Bronfenbrenner in his seminal work posits that human development as a transactional process in which an individual’s development is influenced by interactions with various aspects and spheres of the environment [10]. Extending from Bronfenbrenner’s bioecological theory, Belsky presumed that parenting is multiply determined by three domains: characteristics of the parents, of the child, and of contextual subsystems, such as social support, neighborhood context, and institutional policies [11]. Among these domains, Belsky suggested that the characteristics of parents were the most influential in buffering parent–child interaction, followed by contextual subsystems of support, with child characteristics the least important of the three. He stressed that contextual stress and support can directly affect parenting or indirectly affect parenting by influencing individual psychological well-being, which feeds back to shape parenting. In a similar vein, Furstenberg found that neighborhood context did not provide as great an impact on family or individual outcomes as parental characteristics [12]; nonetheless, neighborhood support and resources had a significant effect as a buffer against mothers’ distress and child abuse.

Franco, Pottick, and Huang used the bioecological theory to examine the effects of neighborhood context on early parenting stress during the first three years of parenthood in the US and to determine whether this was universal across mothers of different races–ethnicities or not [13]. They discovered that neighborhood context was significantly associated with parenting stress and minority parents experience less stress than White parents in higher-disordered (i.e., measured using drug dealing, loitering, gang activity, and disorderly behavior occurrences) neighborhoods [13]. Additionally, Christie-Mizell, Steelman, and Stewart examined mothers between ages 29 and 37 and found that perceived neighborhood disorder was associated with increased maternal psychological distress [14]. They further discovered that mothers’ distress was affected by marital status, educational attainment, household income, and employment [14]. Likewise, substantiated by one-on-one interviews, Wilson discovered that individuals residing in impoverished neighborhoods in the US were more likely to fall prey to a sense of defeatism, hopelessness, and psychological distress [15].

In South Korea (hereafter referred to as Korea), to our knowledge, only one study has empirically examined the association between mothers’ parenting stress and neighborhood characteristics [16]. The study demonstrated that sufficient neighborhood resources and a safe environment were associated with lower parental stress. However, data from this study were gathered in 2009 and were limited to working mothers with children aged one. Cross-sectional designs, in contrast to longitudinal designs, are limited in capturing changes in family function and adaptive self-regulation [17], and literature on child development demonstrates that parental aggression or distress changes significantly over time [18,19]. Additionally, working mothers’ parenting stress is likely to be different from nonworking mothers.

Hence, the goal of the present study was to explore the association between mothers’ parental stress and neighborhood characteristics longitudinally across early childhood (ages 0 to 4) using a nationally representative sample in Korea, including both working and nonworking mothers. In this study, we examined neighborhood characteristics using the following four variables: (1) sufficiency of childcare facilities; (2) convenience to access public recreational spaces and facilities (i.e., playgrounds, parks, walkways); (3) convenience to access cultural facilities (i.e., museums, art galleries, libraries, theaters); and (4) whether it is a good place to raise children in general. The authors hypothesize that mothers residing in neighborhoods with sufficient childcare facilities, convenient to access recreational spaces or facilities, convenient to access cultural facilities, and who perceive their neighborhoods as good places to raise children, report lower parenting stresses than mothers who report otherwise.

## 2. Data and Methods

### 2.1. Data

The data for this study were drawn from the 2008–2012 Panel Study on Korean Children (PSKC) [20]. The PSKC is a nationwide longitudinal study developed to track children’s development over time, along with surrounding characteristics, such as parents’ parenting styles and local neighborhood environments. The survey was conducted by the Korea Institute of Child Care and Education, a government research institute. The PSKC was first conducted from April to July in 2008. A total of 2078 households with newborn babies born in medical institutes nationwide were selected using a multistage random-stratified cluster design to represent the national population. The respondents were revisited every year. Following a total of 2078 households in 2008, approximately 1904 (92%), 1802 (87%), 1754 (84%), and 1703 (82%) completed the survey in 2009, 2010, 2011, and 2012, respectively. Interviews were conducted with the children’s primary caretaker parents (i.e., mother or father); however, if neither parent was available, then interviews were conducted with the caretaker relative (i.e., a person that was a relative by blood, adoption, or marriage, with whom the child was living and who assumed primary responsibility for that child’s care). Therefore, those living in foster homes or institutions were excluded. If the sampled child was separated from the parents or caretaker relatives, then the case was dropped from the survey. Post-stratification weights (also known as non-response weights) were applied to treat for possible drop-out biases, which adjusted for drop-outs by assuming they occur randomly within weighting classes defined by observed variables that were associated with attrition [21]. Post-stratification weights are provided in the PSKC to the users, which are labeled as “longitudinal weights.”

### 2.2. Analytical Sample

In this study, we restricted our sample to children for whom the primary caretaker was a mother and who were born within a marriage. We decided to restrict our sample to children born within a marriage because single mothers may experience higher parenting stress and the number of out-of-wedlock births is minimal in Korea (i.e., 1.9% in 2016, the lowest among OECD countries [22]). In this study, a total of 1536 mothers were examined across four years between children ages zero (0–3 months) to four (48–51 months) on the basis of these criteria.

### 2.3. Measures

#### 2.3.1. Outcome Variables

The outcome variable for this study was mothers’ parenting stress. Parenting stress was measured using a modified version of the Korean Parenting Stress Index, developed by Kim and Kang [23]. In this study, mothers’ parenting stress was examined using ten items measured on a five-point scale (1 = *never*, 2 = *rarely*, 3 = *sometimes*, 4 = *often*, 5 = *always*). The items were as follows: (1) “I am not sure whether I will become a good parent,” (2) “I am concerned as to whether I can raise my child well,” (3) “I feel my child lags behind others because I am not properly performing my roles as a parent,” (4) “I want to escape my child,” (5) “I have difficulty being friendly and warm toward my child,” (6) “I feel confused because there is a lot of childcare-related information to choose from,” (7) “My life is not as enjoyable after having my child,” (8) “I feel burdened by childcare costs,” (9) “I feel bad because it seems to be my fault when my baby appears emotionally unstable,” (10) “I get irritated if my child pesters me to play with him or her when I am tired.” The ten items were averaged to measure mother’s parenting stress and treated as a continuous variable. The Cronbach’s alpha, which measures how coherent a set of items are as an index, was reported as 0.86, indicating a high level of internal consistency within the ten items (relative to a threshold of 0.8). In this study, mothers’ parenting stress was examined at children’s ages zero, one, two, three, and four.

#### 2.3.2. Predictor Variables

The predictor variables for this study were the neighborhood characteristics. In this study, four variables were used to assess the neighborhood characteristics. The PSKC asks respondents whether their neighborhoods (1) have a sufficient number of childcare facilities (i.e., daycare centers, preschools, kindergartens) to which to send their children, (2) are convenient for accessing public recreational spaces or facilities (i.e., playgrounds, parks, walkways), and (3) are convenient for accessing cultural facilities (i.e., museums, art galleries, libraries, theaters), and whether their neighborhoods are (4) good places to raise children in general. All four questions were measured using a five-point Likert scale (1 = very insufficient/very inconvenient/very bad, 2 = insufficient/inconvenient/bad, 3 = neutral, 4 = sufficient/convenient/good, 5 = very sufficient/very convenient/very good). In this study, neighborhood characteristics were treated as ordinal categorical variables.

#### 2.3.3. Control Variables

Demographic information was included as control variables. These encompassed dummy variables for child’s gender (male, female) and unpaid substitute child caretakers who primarily take care of the child instead of parents during daytime weekdays without pay (yes, no), ordinal variables such as childbirth order (first, second, or third or subsequent child), mother’s educational attainment (high school degree or lower, 2-year technical college degree, or 4-year university degree or higher), and child’s age (0, 1, 3, or 4). Each mother’s age, marital satisfaction (5-point Likert scale), satisfaction with spouse’s participation in childcare (5-point Likert scale), and logged household monthly income were measured as continuous variables. Household income was log-transformed to measure percentage changes instead of absolute value changes. Hence, when interpreting the coefficients in the multivariate analyses, a unit change in household income denotes a 1% change in household income. Additionally, we adjusted for inflation before log-transforming to compare real (versus nominal) income values.

### 2.4. Analyses

Multilevel mixed-effects modeling was used to estimate the effects of neighborhood characteristics on the mothers’ parenting stress. Since the PSKC is a longitudinal panel analysis, if we were to compute a pooled ordinal least square regression (OLS) modeling, the results would be biased because of repeated measurements and possible unobserved heterogeneity (i.e., unobserved omitted variables, such as historical trends) [24]. Hence, multilevel mixed-effects modeling was used to control for such biases. Multilevel modeling is one of the most widely used modeling methods to analyze changes over time (i.e., growth curve modeling for longitudinal designs). Multilevel modeling with repeated measures employs the same statistical techniques as multilevel-level modeling with clustered data (i.e., data that are grouped into different clusters, an example being students clustered within schools). In multilevel modeling with repeated data, the measurement occasions (i.e., data points) are nested within cases (e.g., individuals or subjects). Multilevel mixed-effects modeling allowed the researchers to examine the trajectories of mothers’ parenting stress across the children’s ages and to establish how independent factors influenced these trajectories [24]. Mixed-effects modeling assumes that the effects of time-varying variables and time itself are not the same across units. That is, the model assumed that the trajectories of mothers’ parenting stress differed by unit, and thereby, by children’s age. By employing multilevel mixed-effect modeling, we allow the slope (i.e., growth trajectory) and the intercept to vary across individuals, defined as random effects. An alternative popular method of growth curve analysis is latent growth curve modeling using structural equation modeling (SEM). The approach provides the same estimates as the multilevel modeling approach, provided that the model is specified identically in SEM [25]. In this study, we decided to use multilevel modeling instead of latent growth curve modeling because first, adding time-varying or time-invariant covariates to the model is straightforward in multilevel modeling [25] and second, multilevel modeling is preferred when there are many data points [25]. In this study, we employed five data points (children aged zero to four) and we plan to extend the data points to cover mother’s parenting stress until primary school in the future when the data are released. Hence, for these reasons, in this study, multilevel mixed-effects modeling was used instead of latent growth modeling. On the other hand, latent growth modeling is recommended when studying path analyses or when the intercept and slope are used as predictors for other variables [25]. In this study, the software program STATA 16 was used to analyze the models.

## 3. Results

### 3.1. Descriptive Results

**Figure 1:**
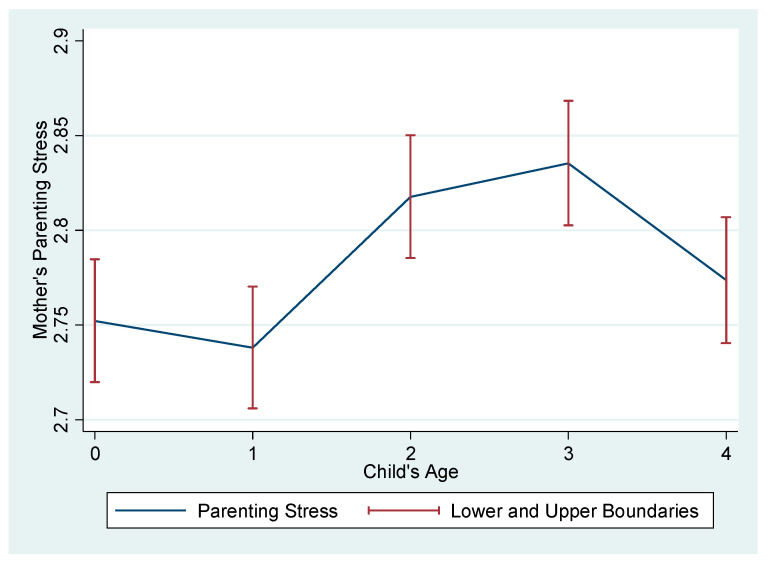
Trajectory of Mother’s Parenting Stress by Child’s Age.

The descriptive results of our sampled mothers and their children, and their neighborhood characteristics, are presented in Table 1. First, with regard to the children’s characteristics, on average, 52% of the children were boys and 49% were girls. The majority of the children were either the first or second child. Second, regarding the mothers’ characteristics, the majority of mothers (45%) had a four-year university degree or higher and were, on average, 31 years old when they gave birth. The mothers’ employment increased proportionate to the child’s age. For example, on average, a mother’s employment rate was 29% when the child was under one year old but rose to 42% by the time the child was four years old. Concurrently, the rate of unpaid caretakers (i.e., a person who primarily takes care of the child instead of the parents during daytime weekdays without pay, such as grandparents) increased from 7% (for children aged 0) to 14% (for children aged 4) during the same period. Mothers were on average 31 years old when the survey was first conducted. Their average monthly household income in 2008 (age 0) was KRW 3,170,000 (inflation adjusted 2005 constant KRW 3,690,000), which was slightly lower than the national household income average of KRW 3,390,000 [26]. However, their income on average rose approximately 135% between 2008 (age 0) to 2012 (age 4), which was higher than the national average of 120% [26]. Overall, our sampled mothers were above “average” satisfied with their marriage and husbands’ participation in childcare. Regarding mothers’ parental stress, the results showed that mothers’ parental stress was highest when the child was three years old. Figure 1 shows that mothers experienced relatively low levels of stress when their children were under one, but that this increased steeply for children aged two to three, before decreasing again for children aged four. Last, regarding neighborhood characteristics, the majority (41–51%) of respondents responded “neutral” to whether their neighborhoods were a “good place to raise children in general”. On average, approximately 28% of respondents reported their neighborhoods had “sufficient” or “very sufficient” childcare facilities (i.e., daycare centers, preschools, kindergartens). Approximately 29% of respondents claimed that it was “convenient” or “very convenient” to access public recreational spaces or facilities (i.e., playgrounds, parks, walkways) in their neighborhoods, and approximately 25% reported that it was “convenient” or “very convenient” to access local cultural facilities (i.e., museums, art galleries, libraries, theaters) in their neighborhoods, respectively.

### 3.2. Bivariate Results

Table 2 presents the bivariate analysis between mothers’ parenting stress and neighborhood characteristics. The results showed that mothers’ parenting stress was significantly (*p* < 0.001) lower when mothers perceived their neighborhoods had sufficiently more childcare facilities. Similarly, mothers who reported that their neighborhoods were more convenient to access public recreational spaces or facilities and cultural facilities experienced significantly (*p* < 0.001) lower parenting stress. Last, mothers who described their neighborhoods as, in general, more “good” places to raise children reported significantly (*p* < 0.001) lower parenting stress. Bonferroni post hoc test was further conducted to investigate which categories significantly differed from the others (i.e., if mother’s mean stress between “neutral” and “good” is significantly different). Bonferroni results showed that overall, the differences between the categories were statistically significant except for the differences between “Sufficient/Convenient/Good” and “Very Sufficient/Very Convenient/Very Good”, represented as “D–E” in Table 3.

### 3.3. Multivariate Results

**Figure 2:**
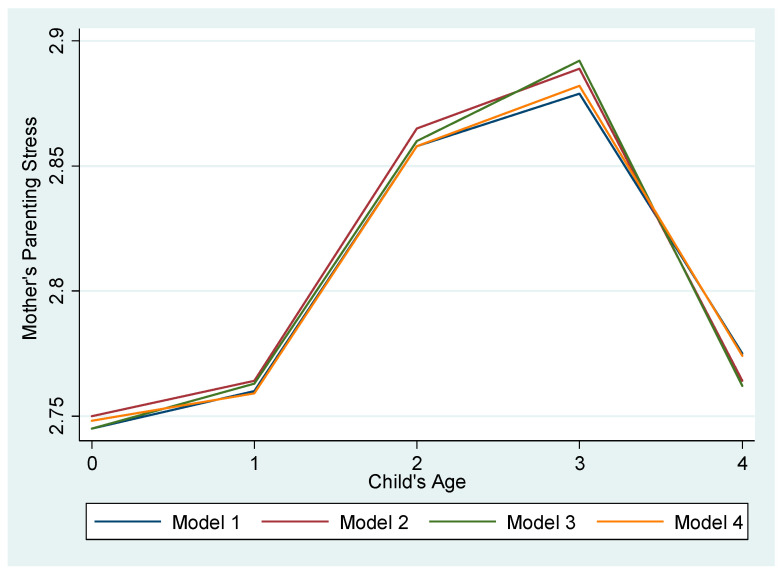
Predicted Mother’s Parenting Stress by Child’s Age. Model 1 represents “Childcare Facilities”, model 2 represents “Public Recreational Spaces or Facilities”, model 3 represents “Cultural Facilities”, and model 4 represents “Good Place to Raise Child in General” from Table 4.

**Figure 3:**
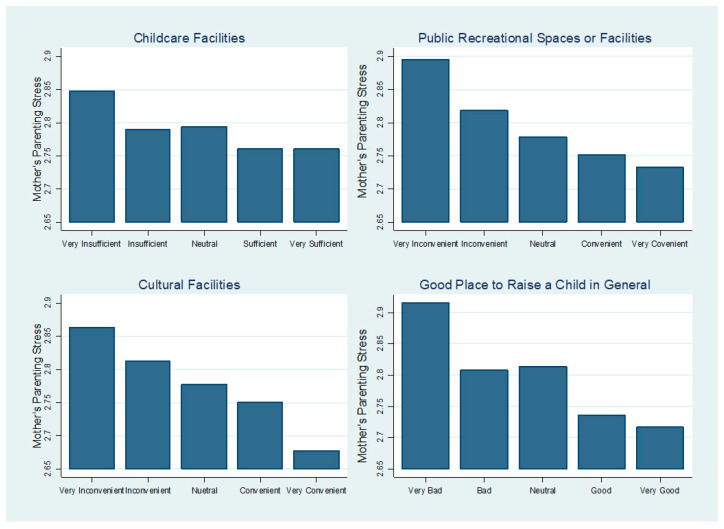
Predicted Mother’s Parenting Stress by Neighborhood Characteristics.

Table 4 presents the multivariate analyses. First, the results showed that after controlling for covariates, mothers’ parenting stress was highest when the child was three years old and lowest when the child was zero years old in all four models. Mother’s parenting stress was significantly higher when the child was two (*b* = 0.11–0.12, *p* < 0.001) and three (*b* = 0.13–0.15, *p* < 0.001) years old compared to when the child was zero years old. Results indicated that mothers’ parenting stress increased steadily from age zero to one, but increased steeply at age two and peaked when children were aged three, before decreasing at aged four in the models (for ease of interpretation, we have calculated and presented the predicted values in Figure 2). Second, with regard to neighborhood effects, the results showed that mothers’ parenting stress was significantly lower when mothers perceived there were “sufficient” (*b* = –0.09, *p* < 0.05)” or “very sufficient” (*b* = –0.09, *p* < 0.05) childcare facilities at their neighborhoods compared to “very insufficient”, even after controlling for demographic characteristics. Likewise, mothers’ parenting stress was significantly lower when respondents reported it was more convenient to access public recreation spaces or facilities at their neighborhoods. The results indicated that mothers’ parenting stress was lower on average 0.08 (*p* < 0.05), 0.12 (*p* < 0.001), 0.14 (*p* < 0.001), and 0.16 (*p* < 0.001) if their neighborhoods were “inconvenient,” “neutral,” “convenient,” and “very convenient” to access public recreational spaces or facilities, in contrast to neighborhoods that were “very inconvenient” to access recreational spaces or facilities, respectively. Additionally, mothers’ parenting stress was significantly lower when mothers perceived their neighborhoods were “neutral” (*b* = –0.09, *p* < 0.01), “convenient” (*b* = –0.11, *p* < 0.001), or “very convenient” (*b* = –0.19, *p* < 0.001) to access cultural facilities compared to when they perceived their neighborhoods were “very inconvenient” to access cultural facilities. Lastly, mothers who reported that their neighborhoods were, in general, “good” (*b* = –0.18, *p* < 0.01) or “very good” (*b* = –0.20, *p* < 0.01) places to raise children experienced significantly lower parenting stress than those who reported their neighborhoods as “very bad” places to raise children. For ease of interpretation, we have graphically presented the results in Figure 3.

**Figure 4:**
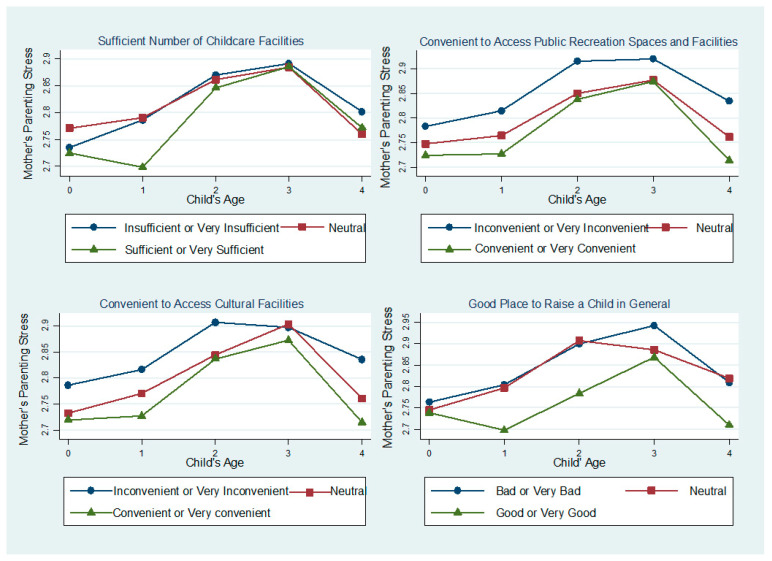
Parenting Stress and Neighborhood Characteristics by Children’s Age.

Further, to understand whether the effect of neighborhood characteristics on mothers’ parenting stress varies by children’s age, we included and examined the interaction terms between neighborhood characteristics and child’s age in the models (Appendix A). To increase the statistical power and ease of interpretation, in the interaction models, we merged “very insufficient” and “insufficient,” and “very sufficient” and “sufficient.” Additionally, we merged “very convenient” and “convenient”, and “very inconvenient” and “inconvenient.” Likewise, “very bad” and “bad,” and “very good” and “good” were each merged. Hence, in the new models, neighborhood characteristics were measured as three-category categorical variables. Results showed that the interaction terms were statistically nonsignificant, indicating that the effects of neighborhood characteristics on mothers’ parenting stress did not vary significantly by children’s age. However, although statistically nonsignificant, the results showed that the difference in mothers’ levels of parenting stress between those who resided in neighborhoods with “sufficient or very sufficient” childcare facilities and those with “insufficient or very insufficient” childcare facilities was greatest when the child was aged one. With regard to public recreational spaces or facilities and cultural facilities, the difference was largest when the child was aged four. Last, the discrepancy in mothers’ parenting stress between those who described their neighborhoods as generally “good or very good” to raise children in and those whose neighborhoods were “bad or very bad” to raise children in was the largest when the children were one and four years old (Figure 4).

## 4. Discussion

The present study examined (1) mothers’ experiences of parenting stress across early childhood (ages 0–4 years) and (2) the association between mothers’ parenting stress and neighborhood characteristics. First, the results showed that mother’s parenting stress increased steeply when children were two years old and was highest when children were three years old but decreased steeply at aged four. Second, mothers who reported their neighborhoods had sufficient childcare facilities, were convenient to access recreational spaces or facilities and cultural facilities, and were good places to raise children exhibited notably lower degrees of parenting stress. Additionally, although statistically nonsignificant, the results indicated that the effects of neighborhoods on mothers’ parenting stress were greatest when children were aged one and four. Childcare facilities (i.e., daycare centers) were especially important when children were one, while public recreational facilities or spaces (i.e., playgrounds, parks, walkways) and cultural facilities (i.e., museums, art galleries, libraries, theaters) were important when children were four years old.

### 4.1. Limitations

Before discussing the implications of this study, it is important to also consider the limitations. First, in this study, neighborhood characteristics were examined based primarily on the physical environment, considering, for example, the sufficiency of childcare centers, public playgrounds, parks, galleries, and libraries. Neighborhood solidarity and support were not examined. Sampson et al. posited that neighborhoods comprise both physical environment and social cohesion [27]. Social cohesion denotes “mutual trust and support among neighbors” [28]. For example, in the context of child maltreatment, neighborhood social cohesion may play a protective role as it provides an informal safety net for parents who are struggling [4,29]. However, as a result of data unavailability, we were unable to examine social cohesion in relation to neighborhood characteristics. Second, the study relied on self-perceived information from respondents. The actual availability of childcare facilities, public recreational spaces or facilities, and cultural facilities may differ from its perceived sufficiency or convenience. For example, some mothers may perceive their neighborhoods to have insufficient childcare facilities even though, in reality, there may be a higher number of facilities than the national average. Third, in this study, we were unable to clarify whether mothers’ perceptions of “sufficient” childcare facilities meant the *actual number of facilities* or the *number of quality facilities*. Further studies are called for that clarify these two concepts. Last, since in this study, we did not lag (i.e., creating variables that are one period behind in time) our predictor neighborhood characteristics, there is also the possibility of reverse causality, in which a mother’s parenting stress may affect perceived neighborhood characteristics. We decided not to lag our predictor variables because there is no information on whether the mother has moved neighborhoods between waves in the PSKC. If a mother has moved neighborhoods between waves, then lagged neighborhood characteristics would represent the previous residing neighborhood instead of the current neighborhood and thus, the estimates between neighborhood characteristics and mothers’ parenting stress would be inaccurate. Additionally, based on Belsky [11] and other previous studies [12,13,14], we assumed that neighborhood characteristics affect parenting stress in this study.

Despite these limitations, this study has notable strengths. First, it is the earliest study to empirically examine how mothers’ parenting stress changes across children’s ages until preschool age at a national level. Second, the study examined the association between mothers’ parenting stress and neighborhood characteristics using several factors: the sufficiency of childcare facilities, public recreational spaces or facilities, and cultural facilities, and respondents’ perceptions of whether their neighborhoods were good places in which to raise children. In addition, to understanding whether the effects of neighborhood characteristics on mothers’ parenting stress are stronger at certain ages of their children, we further examined the effects of neighborhood characteristics on mothers’ parenting stress by children’s age (i.e., the interaction effect).

### 4.2. Implications

Several implications can be drawn from this study. First, the study results showed that when raising children from birth (age 0) to four years old, mothers’ parenting stress was highest when children were aged two to three. As evident from the well-known phrase “the terrible twos”, our results also reflect that mothers experienced a high level of stress during this period. According to Erikson’s stages of child psychosocial development theory [30], children during this period begin to show clear preferences for things. It is the so-called “me do it” stage. For example, a two-year-old child will often insist on choosing clothes, even when the outfits might be inappropriate for the occasion or weather. However, according to Erikson [30], once children reach preschool age (4–6 years), they learn to interact with others, acquire an early sense of moral rules, and imbibe social norms; subsequently, our results also demonstrated that mothers’ parenting stress concurrently decreased as children reached age four. Such findings are consistent with previous studies. Kim et al. examined maternal harsh parenting from birth to age three, and discovered that harsh parenting behavior significantly increased between ages one and two, and was highest at age three [19]. Likewise, Straus and Field examined parental psychological aggression by children ages between infant to seventeen and discovered that the prevalence of psychological aggression increased steeply in ages two to four [31]. The authors denote that while parents consider children up to age one to be limited in their cognitive development, from ages two to four discipline starts. Prendergast and MacPhee examined maternal aggression at children ages three, five, and nine, and found that maternal aggression declined in children between ages five and nine [18]. The Center for Disease Control and Prevention also identifies children younger than four years of age as high-risk factors for child abuse and neglect [32].

Second, our study results demonstrate that neighborhood characteristics play an important role in ameliorating mothers’ parenting stress. According to Belsky’s developmental–ecological theory [11], neighborhoods provide both parents and children with various resources, including social interaction, social support, access to goods and services, and safety. In addition, Sampson et al. posited that shared community and environment help provide a buffer against deviant behaviors and maltreatment, such as child abuse [27]. A study by Prendergast and MacPhee demonstrated that early increases in parenting stress were associated with higher levels of parent aggression in later childhood [18]. Our results indicate that childcare facilities, public recreational spaces and facilities, and cultural facilities help to mitigate mother’s parenting stress in early childhood (ages 0 to 4), which may help prevent parental aggression in the later childhood period. Additionally, in Korea, a lack of childcare facilities, especially quality childcare facilities, has long been cited as one of the main disincentives for having children and as a catalyst for the low total fertility rate. In 2018, Korea’s total fertility rate was 0.98, the lowest among the OECD countries [33]. Sufficient childcare facilities and public recreation and cultural facilities, such as playgrounds, parks, libraries, and museums, help reduce mothers’ stress and may contribute to increasing fertility rates.

Third, we discovered that the effects of neighborhood characteristics on mothers’ parenting stress differed by children’s age. It is important to note that neighborhood characteristics were more salient when children were one and four years old. Sufficiency of childcare facilities was particularly important when the child was one year old, and public recreational spaces or facilities and cultural facilities were particularly important when the child was four years old. Such findings provide important information for policymakers for designing and planning communities. There are currently no studies that have investigated the differential effects of neighborhoods on mother’s parenting stress across children’s age; hence, we call for further future studies to investigate the effects of neighborhood longitudinally extending beyond early childhood to build a more extensive literature on this issue.

## 5. Conclusions

The present study is the first empirical study to examine mothers’ parenting stress trajectory across early childhood in Korea and how neighborhood resources and environment affect mothers’ stress. Consistent with the ecological model, our results also found that neighborhood context played a significant influence on mothers’ psychological wellbeing in Korea. Our results further found that the effects of different neighborhood infrastructures on mothers’ parenting stress differed by children’s age, where childcare facilities were particularly important when children were one year old, and public recreational spaces or facilities and cultural facilities were particularly important when children were four years old.

## Figures and Tables

**Table 1:** Descriptive Statistics for All Study Variables by Child’s Age (*n* = 1536).

Variables	Age 0	Age 1	Age 2	Age 3	Age 4
	*n* (%)				
**Child’s Characteristics**					
Child’s gender ^1^					
Male	784 (51.5%)	784 (51.5%)	784 (51.5%)	784 (51.5%)	784 (51.5%)
Female	752 (48.5%)	752 (48.5%)	752 (48.5%)	752 (48.5%)	752 (48.5%)
Child’s birth order					
First	707 (46.0%)	703 (45.8%)	703 (45.8%)	703 (45.8%)	703 (45.8%)
Second	649 (42.3%)	653 (42.5%)	653 (42.5%)	652 (42.4%)	652 (42.4%)
Third or subsequent	180 (11.7%)	180 (11.5%)	180 (11.5%)	181 (11.7%)	181 (11.7%)
**Mother’s and Household Characteristics**					
Mother’s educational attainment					
High school degree or lower	468 (30.5%)	458 (29.8%)	444 (28.9%)	444 (28.9%)	444 (28.9%)
2-year technical college degree	458 (29.8%)	403 (26.3%)	401 (26.1%)	401 (26.1%)	401 (26.1%)
4-year university degree or higher	609 (39.7%)	674 (43.9%)	691 (45.0%)	691 (45.0%)	691 (45.0%)
Mother’s employment status					
Employed ^2^ or studying	445 (29.0%)	481 (31.3%)	508 (33.1%)	614 (40.0%)	650 (42.3%)
Unemployed or economically inactive	1091 (71.0%)	1055 (68.7%)	1028 (66.9%)	921 (60.0%)	886 (57.7%)
Unpaid substitute caretaker ^3^					
Access to	104 (6.8%)	104 (6.8%)	101 (6.6%)	101 (6.6%)	211 (13.8%)
No access to	1432 (93.2%)	1432 (93.2%)	1434 (93.4%)	1434 (93.4%)	1324 (86.2%)
**Mean (Standard Error)**					
Mother’s age	31.3 (0.1)	32.1 (0.1)	32.8 (0.1)	33.8 (0.1)	34.8 (0.1)
Household monthly income (KRW 10,000) ^4^	369.1 (4.5)	471.7 (18.1)	417.9 (11.2)	556.6 (23.8)	498.1 (12.7)
Marital satisfaction (1: not satisfied–5: very satisfied)	3.8 (2.0 × 10^−2^)	3.9 (2.0 × 10^−2^)	3.8 (2.0 × 10^−2^)	3.7 (2.0 × 10^−2^)	3.7 (2.0 × 10^−2^)
Husband’s childcare satisfaction (1: not satisfied–5: very satisfied)	3.6 (2.0 × 10^−2^)	3.6 (2.0 × 10^−2^)	3.7 (2.0 × 10^−2^)	3.6 (2.0 × 10^−2^)	3.6 (2.0 × 10^−2^)
Mother’s parenting stress level (1: never–5: always)	2.75 (1.7 × 10^−2^)	2.74 (1.6 × 10^−2^)	2.82 (1.7 × 10^−2^)	2.84 (1.7 × 10^−2^)	2.77 (1.7 × 10^−2^)
**Neighborhood Characteristics**					
Sufficient childcare facilities (i.e., daycare centers, preschools)					
Very insufficient	65 (4.2%)	75 (4.9%)	58 (3.8%)	108 (7.02%)	92 (6.0%)
Insufficient	263 (17.1%)	327 (21.3%)	310 (20.2%)	399 (26.0%)	290 (18.9%)
Neutral	511 (33.3%)	665 (43.3%)	644 (41.9%)	684 (44.5%)	571 (37.2%)
Sufficient	579 (37.7%)	390 (25.4%)	422 (27.5%)	301 (19.6%)	479 (31.2%)
Very sufficient	118 (7.7%)	78 (5.1%)	101 (6.6%)	92 (2.9%)	103 (6.7%)
Convenient to access public recreational spaces or facilities (i.e., playgrounds, parks, walkways)					
Very inconvenient	84 (5.5%)	109 (7.1%)	91 (5.9%)	121 (7.9%)	192 (12.5%)
Inconvenient	329 (21.4%)	321 (20.9%)	281 (18.3%)	286 (18.6%)	355 (23.1%)
Neutral	373 (24.3%)	435 (28.3%)	468 (30.5%)	485 (31.6%)	525 (34.2%)
Convenient	587 (78.2%)	548 (35.7%)	554 (36.1%)	567 (36.9%)	372 (24.2%)
Very convenient	164 (10.7%)	123 91(8.0%)	141 (9.2%)	77 (5.0%)	92 (6.0%)
Convenient to access cultural facilities (i.e., museums, libraries, art galleries, theaters)					
Very inconvenient	154 (10.0%)	209 (13.6%)	161 (10.5%)	169 (11.0%)	292 (19.0%)
Inconvenient	218 (14.2%)	183 (11.9%)	177 (11.5%)	194 (12.6%)	229 (14.9%)
Neutral	373 (24.3%)	432 (28.1%)	464 (30.2%)	487 (31.7%)	536 (34.9%)
Convenient	717 (46.7%)	677 (44.1%)	688 (44.8%)	664 (43.2%)	473 (30.8%)
Very convenient	74 (4.8%)	35 (2.3%)	46 (3.0%)	23 (1.5%)	5 (0.3%)
Good place to raise child in general					
Very bad	31 (2.0%)	37 (2.4%)	26 (1.7%)	35 (2.3%)	38 (2.5%)
Bad	247 (16.1%)	207 (13.5%)	22 (14.2%)	178 (11.6%)	198 (12.9%)
Neutral	632 (41.2%)	737(48.0%)	743 (48.4%)	784 (51.1%)	680 (44.3%)
Good	552 (36.0%)	493 (32.1%)	494 (32.2%)	507 (33.0%)	522 (34.0%)
Very good	77 (5.0%)	61 (4.0%)	52 (3.4%)	32 (2.1%)	95 (6.2%)

Notes: Survey weights were applied to represent the population parameter. ^1^ Time-invariant variable, constant across age. ^2^ Including part-time employment and those on leave. ^3^ Those who primarily take care of the child instead of the parents during daytime weekdays without pay, such as grandparents. ^4^ Inflation adjusted household income (constant 2015 KRW).

**Table 2:** Mean Mothers’ Parenting Stress by Neighborhood Characteristics (*n* = 1536).

Neighborhood Characteristics	Childcare Facilities	Public Recreational Space or Facilities	Cultural Facilities	Good Place to Raise Child in General
Very Insufficient/Very Inconvenient/Very Bad ^1^	2.99	2.97	2.93	3.16
Insufficient/Inconvenient/Bad ^1^	2.88	2.86	2.86	2.93
Neutral	2.79	2.78	2.79	2.82
Sufficient/Convenient/Good ^1^	2.73	2.75	2.74	2.71
Very Sufficient/Very Convenient/Very Good ^1^	2.75	2.69	2.58	2.68
ANOVA *F*(*df*)	21.28 (4) ***	21.84 (4) ***	18.63 (4) ***	35.90 (4) ***

Notes: Values represent mother’s parenting stress levels (ranging from 1 to 5). Higher values indicate higher stress levels. Survey weights were applied to represent the population parameters. ^1^ “Childcare Facilities” corresponds with sufficiency, “Public Recreational Space or Facilities” and “Cultural Facilities” correspond with convenience, and “Good Place to Raise Child in General” corresponds with good and bad. *** *p* < 0.001.

**Table 3:** Bonferroni Post Hoc Test Results.

Category Difference	Childcare Facilities	Public Recreational Space or Facilities	Cultural Facilities	Good Place to Raise Child in General
A–B ^1^	0.11 **	0.11	0.07	0.23 ***
A–C ^1^	0.20 ***	0.19 ***	0.14 ***	0.34 ***
A–D ^1^	0.26 ***	0.22 ***	0.19 ***	0.45 ***
A–E ^1^	0.24 ***	0.26 ***	0.25 ***	0.48 ***
B–C ^1^	0.09 *	0.08 *	0.07	0.11 *
B–D ^1^	0.15 ***	0.11 ***	0.12 ***	0.22 ***
B–E ^1^	0.13 **	0.17 ***	0.28 ***	0.25 ***
C–D ^1^	0.06 ***	0.03	0.05 *	0.11 ***
C–E ^1^	0.04	0.09 ***	0.21 *	0.14 ***
D–E ^1^	–0.02	0.06	0.16	0.03

Notes: Values represent mean mother’s parenting stress difference between categories, calculated based on Table 2. ^1^ “A” represents category “very insufficient/very inconvenient/very bad”; “B” represents category “insufficient/inconvenient/bad”; “C” represents category “neutral”; “D” represents” “sufficient/convenient/good”; and “E” represents category “very sufficient/very convenient/very good.” * *p* < 0.05. ** *p* < 0.01, *** *p* < 0.001.

**Table 4:** Multilevel Mixed Effect Modeling (*n* = 1536).

	Childcare Facilities	Public Recreational Spaces or Facilities	Cultural Facilities	Good Place to Raise Child in General
	Coefficient (Standard Error)	Coefficient (Standard Error)	Coefficient (Standard Error)	Coefficient (Standard Error)
**Fixed effects**				
Neighborhood Characteristics (ref: very insufficient/very inconvenient/very bad) ^1^				
Insufficient/inconvenient/bad	−0.06 (0.04)	−0.08 (0.03) *	−0.05 (0.03)	−0.11 (0.06)
Neutral	−0.05 (0.04)	−0.12 (0.03) ***	−0.09 (0.02) **	−0.10 (0.06)
Sufficient/convenient/good	−0.09 (0.04) *	−0.14 (0.03) ***	−0.11 (0.03) ***	−0.18 (0.06) **
Very Sufficient/very convenient/very good	−0.09 (0.05) *	−0.16 (0.04) ***	−0.19 (0.06) **	−0.20 (0.06) **
Child’s age (ref: age 0)				
Age 1	0.01 (0.02)	0.01 (0.02)	0.02 (0.02)	0.01 (0.02)
Age 2	0.11 (0.02) ***	0.12 (0.02) ***	0.11 (0.02) ***	0.11 (0.02) ***
Age 3	0.13 (0.03) ***	0.14 (0.03) ***	0.15 (0.03) ***	0.13 (0.03) ***
Age 4	0.03 (0.02)	0.01 (0.02)	0.02 (0.02)	0.03 (0.02)
Female Child	−0.04 (0.03)	−0.04 (0.03)	−0.04 (0.03)	−0.03 (0.03)
Mother’s age	−0.01 (3.93 × 10^−2^)	−0.01 (3.92 × 10^−2^)	−0.01 (3.95 × 10^−2^)	−0.01 (3.91 × 10^−2^)
Child’s birth order (ref: first)				
Second	0.12 (0.03) ***	0.12 (0.03) ***	0.12 (0.03) ***	0.12 (0.03) ***
Third or subsequent	−0.02 (0.05)	−0.03 (0.05)	−0.03 (0.05)	−0.02 (0.05)
Mother’s Education (ref: high school degree or lower)				
2-year technical college degree	−0.09 (0.03) **	−0.08 (0.03) **	−0.08 (0.03) **	−0.08 (0.03) **
4-year university degree or higher	−0.11(0.03) ***	−0.10(0.03) ***	−0.09 (0.03) **	−0.10 (0.03) ***
Marital satisfaction	−0.16 (0.01) ***	−0.16 (0.01) ***	−0.16 (0.01) ***	−0.16 (0.01) ***
Spouse participation in childcare satisfaction	−0.08 (0.01) ***	−0.08 (0.01) ***	−0.08 (0.01) ***	−0.08 (0.01) ***
Unpaid substitute caretaker	7.87 × 10^−4^ (0.03)	−4.94 × 10^−4^ (0.03)	−3.93 × 10^−4^ (0.03)	9.71 × 10^−5^ (0.03)
Logged monthly household income	−0.03 (0.02) *	−0.03 (0.02)	−0.03 (0.02)	−0.03 (0.02)
Constant	4.21 (0.16) ***	4.20 (0.16) ***	4.20 (0.16) ***	4.23 (0.16) ***
**Random effects**				
Standard deviation (age)	0.09 (0.01)	0.09 (0.01)	0.09 (0.01)	0.09 (0.01)
Standard deviation (constant)	0.52 (0.02)	0.51 (0.02)	0.51 (0.02)	0.51 (0.02)
Correlation (age, constant)	−0.44 (0.05)	−0.44 (0.05)	−0.42 (0.05)	−0.42 (0.05)
Standard deviation (residual)	0.36 (0.01)	0.36 (0.01)	0.36 (0.01)	0.36 (0.01)

Notes: ^1^ “Childcare Facilities” corresponds with sufficiency, “Public Recreational Space or Facilities” and “Cultural Facilities” correspond with convenience, and “Good Place to Raise Child in General” corresponds with good and bad. * *p* < 0.05. ** *p* < 0.01, *** *p* < 0.001.

## Data Availability

Publicly available datasets were analyzed in this study. This data can be found here: https://panel.kicce.re.kr/panel/module/rawDataManage/index.do?menu_idx=56 (accessed on 5 March 2021).

## Appendix A

**Table A1:** Multilevel Mixed Effect Modeling Including Interaction Terms (*n* = 1536).

	Childcare Facilities	Public Recreational Spaces or Facilities	Cultural Facilities	Good Place to Raise Child in General
	Coefficient (Standard Error)	Coefficient (Standard Error)	Coefficient (Standard Error)	Coefficient (Standard Error)
**Fixed effects**				
Neighborhood Characteristics (ref: Insufficient/Inconvenient/Bad)				
Neutral	0.03 (0.04)	−0.05 (0.04)	−0.07 (0.04)	−0.04 (0.04)
Sufficient/Convenient/Good	−0.01 (0.04)	−0.08 (0.03) *	−0.08 (0.03) *	−0.04 (0.04)
Child’s age (ref: age 0)				
Age 1	0.05 (0.03)	0.01 (0.03)	0.01 (0.03)	0.02 (0.04)
Age 2	0.14 (0.04) **	0.11 (0.04) **	0.10 (0.04) *	0.12 (0.05) *
Age 3	0.14 (0.05) **	0.14 (0.06) *	0.14 (0.07) *	0.20 (0.08) *
Age 4	0.07 (0.04)	0.03 (0.03)	0.04 (0.04)	0.04 (0.05)
Neighborhood * Child’s Age				
Neutral × Age 1	−0.02 (0.05)	0.02 (0.05)	0.03 (0.05)	0.04 (0.05)
Sufficient/Convenient/Good × Age 1	−0.09 (0.04) *	−0.01 (0.04)	1.43 × 10^−3^ (0.04)	−0.05 (0.05)
Neutral × Age 2	−0.06 (0.05)	−0.03 (0.04)	−0.01 (0.06)	0.04 (0.06)
Sufficient/Convenient/Good × Age 2	−0.03 (0.07)	−2.48 × 10^−3^ (0.05)	0.01 (0.05)	−0.08 (0.06)
Neutral × Age 3	−0.04 (0.08)	−0.02 (0.08)	0.03 (0.09)	−0.06 (0.09)
Sufficient/Convenient/Good × Age 3	−2.18 × 10^−4 ^(0.09)	−0.02 (0.07)	−0.01 (0.08)	−0.10 (0.09)
Neutral × Age 4	−0.08 (0.05)	−0.03 (0.05)	−0.02 (0.05)	0.03 (0.05)
Sufficient/Convenient/Good × Age 4	−0.03 (0.05)	0.04 (0.04)	−0.04 (0.05)	−0.07 (0.05)
Female Child	−0.03 (0.02)	−0.03 (0.03)	−0.04 (0.02)	−0.03 (0.02)
Mother’s age	−0.01 (3.44 × 10^−3^) *	−0.01 (3.45 × 10^−3^) *	−0.01 (3.49 × 10^−3^) *	−0.01 (3.44 × 10^−3^)
Child’s birth order (ref: first)				
Second	0.13 (0.03) ***	0.12 (0.03) ***	0.12 (0.03) ***	0.13 (0.03) ***
Third or subsequent	3.58 × 10^−3^ (0.04)	−2.01 × 10^−3^ (0.04)	4.42 × 10^−3^ (0.04)	−0.02 (0.05)
Mother’s Education (ref: high school degree or lower)				
2-year technical college degree	−0.07 (0.03) *	−0.07 (0.03) *	−0.07 (0.03) **	−0.06 (0.03) **
4-year university degree or higher	−0.10 (0.03) ***	−0.09 (0.03) **	−0.08 (0.03) **	−0.08(0.03) **
Marital satisfaction	−0.16 (0.01) ***	−0.16 (0.01) ***	−0.17 (0.01) ***	−0.16 (0.01) ***
Spouse participation in childcare satisfaction	−0.07 (0.01) ***	−0.08 (0.01) ***	−0.07 (0.01) ***	−0.07 (0.01) ***
Unpaid substitute caretaker	3.92 × 10^−3^(0.03)	4.71 × 10^−3^(0.03)	0.01 (0.03)	3.86 × 10^−3^ (0.03)
Logged monthly household income	−0.04 (0.01) *	−0.04 (0.01) *	−0.04 (0.02) *	−0.04 (0.01) *
Constant	4.16 (0.15) ***	4.16 (0.14) ***	4.19 (0.15) ***	4.15 (0.15) ***
**Random effects**				
Standard deviation (age)	0.09 (0.01)	0.09 (0.01)	0.09 (0.01)	0.09 (0.01)
Standard deviation (constant)	0.51 (0.02)	0.51 (0.02)	0.50 (0.02)	0.51 (0.02)
Correlation (age, constant)	−0.45 (0.05)	−0.45 (0.05)	−0.42 (0.05)	−0.44 (0.05)
Standard deviation (residual)	0.35 (0.01)	0.36 (0.01)	0.36 (0.01)	0.35 (0.01)

Notes: We merged “very insufficient/very inconvenient/very bad” and “insufficient/inconvenient/bad” into one category, and “very sufficient/very convenient/very good” and “sufficient/convenient/good” into another category. “Childcare Facilities” corresponds with sufficiency, “Public Recreational Space or Facilities” and “Cultural Facilities” correspond with convenience, and “Good Place to Raise Child in General” corresponds with good and bad * *p* < 0.05. ** *p* < 0.01, *** *p* < 0.001.

## References

[1] Pinderhughes E.E., Nix R., Foster E.M., Jones D. (2001). Parenting in context: Impact of neighborhood poverty, residential stability, public services, social networks, and danger on parental behaviors. J. Marriage Fam..

[2] Brooks-Gunn J., Duncan G.J., Aber J.L. (1997). Neighborhood Poverty: Context and Consequences for Children.

[3] Coulton C.J., Crampton D.S., Irwin M., Spilsbury J.C., Korbin J.E. (2007). How neighborhoods influence child maltreatment: A review of the literature and alternative pathways. Child Abuse Negl..

[4] Maguire-Jack K., Showalter K. (2016). The protective effect of neighborhood social cohesion in child abuse and neglect. Child Abuse Negl..

[5] Leventhal T., Brooks-Gunn J. (2000). The neighborhoods they live in: The effects of neighborhood residence on child and adolescent outcomes. Psychol. Bull..

[6] McPherson A.V., Lewis K.M., Lynn A.E., Haskett M.E., Behrend T.S. (2009). Predictors of parenting stress for abusive and nonabusive mothers. J. Child Fam. Stud..

[7] Crnic K.A., Lows C., Bornstein M.H. (2002). Everyday stresses and parenting. Handbook of Parenting. Practical Issues in Parenting.

[8] Crnic K.A., Greenberg M.T. (1990). Minor parenting stresses with young children. Child Dev..

[9] Maguire-Jack K., Wang X. (2016). Pathways from neighborhood to neglect: The mediating effects of social support and parenting stress. Child. Youth Serv. Rev..

[10] Bronfenbrenner U. (1979). The Ecology of Human Development: Experiments by Nature and Design.

[11] Belsky J. (1984). The determinants of parenting: A process model. Child Dev..

[12] Furstenberg F.F. (2001). Managing to make it: Afterthoughts. J. Fam. Issues.

[13] Franco L.M., Pottick K.J., Huang C. (2010). Early parenthood in a community context: Neighborhood conditions, race-ethnicity, and parenting stress. J. Community Psychol..

[14] Christie-Mizell C.A., Steelman L.C., Stewart J. (2003). Seeing their surroundings: The effects of neighborhood setting and race on maternal distress. Soc. Sci. Res..

[15] Wilson W.J. (1999). When Work Disappears: The World of the New Urban Poor.

[16] Hong Y.-J., Lee K. (2019). The effect of parenting stress on social interactive parenting with a focus on Korean employed mothers’ parenting support from ecological contexts. Child. Youth Serv. Rev..

[17] Macphee D., Lunkenheimer E., Riggs N. (2015). Resilience as regulation of developmental and family processes. Fam. Relat..

[18] Prendergast S., Macphee D. (2020). Trajectories of maternal aggression in early childhood: Associations with parenting stress, family resources, and neighborhood cohesion. Child Abuse Negl..

[19] Kim H., Pears K.C., Fisher P.A., Connelly C.D., Landsverk J.A. (2010). Trajectories of maternal harsh parenting in the first three years of life. Child Abuse Negl..

[20] Korea Institute of Child Care and Education Panel Study on Korean Children. https://panel.kicce.re.kr/engpanel/index.do.

[21] Henderson M., Hillygus D., Tompson T. (2010). “Sour grapes” or rational voting? Voter decision making among thwarted primary voters in 2008. Public Opin. Q..

[22] OECD Family Database (2016). SF2.4 Share of Births Outside of Marriage. http://www.oecd.org/els/family/database.htm.

[23] Kim K., Kang H. (1997). Development of the Parenting Stress Scale. Fam. Environ. Res..

[24] Wooldridge J.M. (2008). Introductory Econometrics: A Modern Approach.

[25] Hox J., Stoel R.D. (2014). Multilevel and SEM Approached to Growth Curve Modeling. Wiley Stats Ref. Stat. Ref. Online.

[26] Statistics Korea Monthly Average Household Income. https://kosis.kr/statHtml/statHtml.do?orgId=101&tblId=DT_1L9H008&vw_cd=MT_ZTITLE&list_id=G_A_4_1_1&seqNo=&lang_mode=ko&language=kor&obj_var_id=&itm_id=&conn_path=MT_ZTITLE.

[27] Sampson R.J., Raudenbush S.W., Earls F. (1997). Neighborhoods and violent crime: A multilevel study of collective efficacy. Science.

[28] Berkman L.F., Kawachi I., Berkman L.F., Kawachi I., Glymour M.M. (2015). Social cohesion, social capital, and health. Social Epidemiology.

[29] Freisthler B., Maguire-Jack K. (2015). Understanding the interplay between neighborhood structural factors, social processes, and alcohol outlets on child physical abuse. Child Maltreat..

[30] Erikson E.H. (1963). Childhood and Society.

[31] Straus M.A., Field C.J. (2003). Psychological aggression by American parents: National data on prevalence, chronicity, and severity. J. Marriage Fam.

[32] Center for Disease Control and Prevention Child Abuse and Neglect: Risk and Protective Factors. https://www.cdc.gov/violenceprevention/childabuseandneglect/riskprotectivefactors.html.

[33] OECD Family Database (2018). SF2.1 Fertility Rates. http://www.oecd.org/els/family/database.htm.

